# Liquid Biopsy in Oral Cancer

**DOI:** 10.3390/ijms19061704

**Published:** 2018-06-08

**Authors:** Fatima Lousada-Fernandez, Oscar Rapado-Gonzalez, Jose-Luis Lopez-Cedrun, Rafael Lopez-Lopez, Laura Muinelo-Romay, Maria Mercedes Suarez-Cunqueiro

**Affiliations:** 1 Department of Surgery and Medical Surgical Specialties, Medicine and Dentistry School, Universidade de Santiago de Compostela, 15782 Spain; fatimalousadafernandez@gmail.com (F.L.-F.); oscarr16691@gmail.com (O.R.-G.); 2 Liquid Biopsy Analysis Unit, Translational Medical Oncology (Oncomet), Health Research Institute of Santiago de Compostela (IDIS), CIBERONC, 15706 Santiago de Compostela, Spain; 3 Department of Oral and Maxillofacial Surgery, Complexo Hospitalario Universitario de A Coruña (SERGAS), 15006 La Coruña, Spain; lopezcedrun@centromaxilofacial.com; 4 Translational Medical Oncology, Health Research Institute of Santiago de Compostela (IDIS), Complexo Hospitalario Universitario de Santiago de Compostela (SERGAS), ONCOMET, 15706 Santiago de Compostela, Spain; rafa.lopez.lopez@gmail.com; 5 Oral Sciences, Health Research Institute of Santiago de Compostela (IDIS), 15706 Santiago de Compostela, Spain

**Keywords:** oral cancer, circulating tumour cells (CTCs), circulating tumour DNA (ctDNA), exosomes, liquid biopsy

## Abstract

Oral cancer is one of the most prevalent forms of cancer worldwide. Carcinogenesis is a complex process, in which heterogeneity plays an important role in the development and progression of the disease. This review provides an overview of the current biological and clinical significance of circulating tumour cells (CTCs), circulating tumour DNA (ctDNA), and exosomes for diagnosis and prognosis of oral cancer. We highlight the importance of liquid biopsy—using blood and saliva—which represents a potential alternative to solid biopsy for diagnosis and prognosis. Moreover, liquid biomarkers allow for the real-time monitoring of tumour evolution and therapeutic responses, initiating the era of personalized medicine. However, in oral cancer, the impact of liquid biopsies in clinical settings is still limited, requiring further studies to discover the best scenario for its clinical use.

## 1. Introduction

Oral cancer is one of the most prevalent forms of cancers worldwide, showing an incidence and mortality twice as high in men (2.3% and 1.7%, respectively) compared to women (1.2% and 0.8%, respectively) [1]. It is responsible for 145,000 deaths worldwide and an annual incidence of 300,000 diagnosed cases, being a serious and growing problem in less developed regions especially [2]. Oral cancer is a subset of head and neck cancer that includes cancers of the mucosal surfaces of the lips, floor of the mouth, front two-thirds of the tongue, buccal mucosa, lower and upper gingival surfaces, hard palate, soft palate, and retromolar trigone [3,4]. This cancer is characterized by a multifactorial etiology and its development has been associated with several risk factors such as tobacco smoking, alcohol consumption [5], human papillomavirus (HPV) [6], pesticides [7], presence of potential malignant oral lesions [8], weakened immune system, diet with nutritional deficiencies, hereditary predisposition, or radiation [9,10]. Oral carcinogenesis, characterized by several genetic disorders, is a complex multistep process that disturbs cell signaling, growth, survival, motility, angiogenesis, and cell cycle control [11]. Oncogenes and tumour suppressor genes such as *CCND1*, *EGFR*, *RAS*, *VEGF*, *p53*, *CDKN2A*, *STAT3*, and *Rb* have been implicated in oral cancer [11,12,13]. Oral cancer is preventable and curable in early stages, but the majority of cases of oral squamous cell carcinomas (OSCC) are not diagnosed until advanced stages, a point at which therapy is less effective and the prognosis is worse [14,15]. Tissue biopsy is the gold standard in the diagnosis of oral cancer, but it is invasive, costly, and time-consuming, and it is potentially harmful. Moreover, conventional biopsies are temporally and spatially limited and often provide a brief snapshot of a single region of a heterogeneous tumour [16].

Today, it is well known that the molecular profiles of tumours have dynamic behavior over time [17,18]. Carcinogenesis is a complex process in which heterogeneity plays an important role in the development and progression. Genetic, transcriptomic, epigenetic, and/or phenotypic changes could explain inter- and intratumoural heterogeneity. Thus, cellular mosaicism is closely related to carcinogenesis, resistance to therapy, and the metastasis capacity of distinct subpopulations of cancer cells [19], knowledge of which is essential for an effective therapy.

Nowadays, research efforts are focused on the discovery of new, noninvasive methods for the diagnosis and comprehension of the tumour genomic architecture to monitor tumour evolution and therapeutic response in real time [18,20]. In this sense, the field of liquid biopsy has emerged as a revolution in multiple areas of oncology and the development of tumour-precision medicine. Liquid biopsy is as a noninvasive diagnostic tool, based on the detection of circulating tumour cells (CTCs), circulating tumour DNA (ctDNA) and circulating tumour RNA (ctRNA), proteins, and exosomes [18,21,22]. Importantly, in addition to blood, there are other bodily fluids such as urine, saliva, seminal plasma, pleural effusions, cerebrospinal fluid, sputum, and stool samples that can be used for a liquid biopsy [23]. One of the key advantages of studying liquid biopsies is that they provide a personalized snapshot of primary and metastatic tumours at successive time points, providing knowledge of the tumour burden so as to detect early evidence of recurrence or resistance to the disease [24] and helping clinicians in their therapeutic decision-making [21,25]. Therefore, by using liquid biopsies, we can obtain a molecular profile for each patient. Different tumour subtypes could complement the tumor, node, metastasis (TNM) staging system. The TNM staging system was established in 1968 and it is based on primary tumour size, lymph node involvement, and presence of distant metastasis. The current eighth edition TNM classification includes some new changes of head and neck cancer, such as HPV/p16 in oropharyngeal cancer and modification of the T category, including depth of invasion for OSCC [26]. However, given that the staging system does not reflect the biological heterogeneity of a tumour, an appropriate addition to this staging system would be the inclusion of the molecular type. This will help to create more highly personalized medicine, decreasing the risk of overtreatment or undertreatment [27].

**Figure 1:**
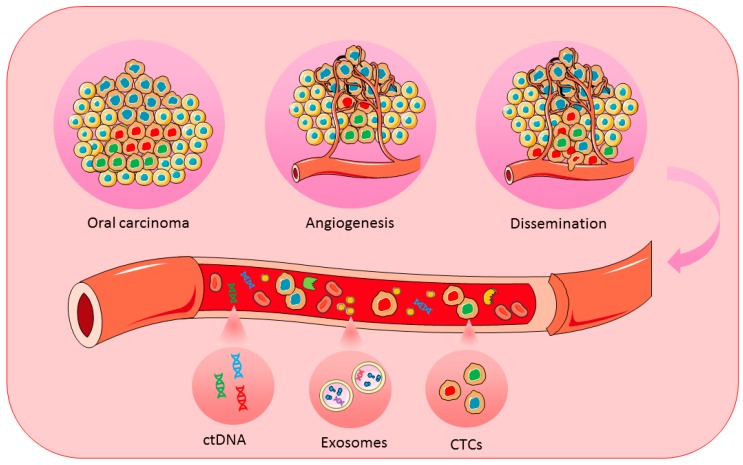
Schematic representation of CTCs, ctDNA, and exosomes for achieving personalized medicine in oral cancer. CTCs, circulating tumour cells; ctDNA, circulating tumour DNA.

This review provides an overview regarding the current biological and clinical significance of CTCs, ctDNA, and exosomes for the diagnosis and prognosis of oral cancer (Figure 1).

## 2. Blood Markers for Oral Cancer

### 2.1. Circulating Tumour Cells (CTCs)

Metastasis is the result of the dissemination of primary tumour cells by entering the bloodstream and reaching a different location, where a new tumour is formed [28]. Importantly, it is responsible for 90% of the mortality of cancer patients [29]. CTCs are cells released by the primary tumour or by distant metastatic lesions into the bloodstream, so they share most of the mutational profile with tumoural clones present in the primary tumour [30]. They can circulate alone or form clusters that have a greater metastatic potential [29]. In order to get into circulation and form metastases, they must undergo a multistep process called the “metastatic cascade”, which includes their epithelial–mesenchymal transition (EMT), intravasation, survival in an adverse environment, and their extravasation at distant sites where the mesenchymal–epithelial inverse transformation (MET) takes place [31,32,33]. EMT occurs in epithelial cells, which develop mesenchymal-like properties, including the downregulation of epithelial markers [34]. Accordingly, the appearance of CTCs with a more epithelial phenotype have been associated with those with a higher ability to form metastases [35].

One of the characteristics of CTCs is that they are in low abundance, with only approximately 1 CTC per 10^7^ white blood cells per milliliter of blood in metastatic patients [36]. Numerous techniques for CTC enrichment and detection have emerged during the last decades based on antigen-dependent and -independent techniques [37]. CellSearch System (Menarini, Bologna, Italy) is the most common technique used to capture and enumerate CTCs and the only United States Food and Drug Administration (FDA)-approved platform for a prognostic use in breast, prostate, and colorectal cancer [38]. The CellSearch platform isolates CTCs from whole blood based on the positive expression of the epithelial marker EpCAM (epithelial cell adhesion molecule); cytokeratins 8, 18 and 19; and the negative expression of CD45 [39]. Other methods for CTC enrichment use the size, electrical charge, density, or deformability [40]. These methods that depend on the physical properties of CTCs for their capture usually show high recovery efficiency, greater than 80%. Nevertheless, we must keep in mind that not all CTCs are larger than nucleated blood cells; for example, tumour cells undergoing apoptosis or those in EMT may be smaller in size [41].

Independent of the isolation technique employed, the quantification and the genomic characterization of CTCs have proven to be useful for differential diagnosis, prognosis, cancer recurrence detection, and real-time monitoring of tumour evolution and therapeutic efficacy in different tumours [42,43]. Oral cancer studies have been mainly focused on the validation of CTCs as a prognostic and recurrence predictor, taking into account only CTC levels [43,44], only qualitative characteristics [30,41,42,43,44,45,46,47,48], or both [35,49].

In 2003, Partridge et al. [50] evaluated the levels of disseminated tumour cells (DTCs) preoperatively and intraoperatively in both blood and bone marrow from 40 OSCC patients. They found a high risk of loco-regional recurrence and distant metastasis associated with the presence of DTCs. In addition, their presence was correlated with lower distant-metastasis-free-survival and disease-free survival rates. Gröbe et al. [44] also studied the prognostic relevance of CTC counting using the CellSearch system in 110 patients in various OSCC stages, correlating their levels with clinicopathological parameters and rate of recurrence and death. They also analyzed DTCs in bone marrow at the iliac crest. They found DTCs in 20% of patients, while CTC presence was found to be 12.5% with a range of 1–14 CTCs/7.5 mL. CTC detection was significantly correlated with tumour size, whereas disseminated DTCs were significantly correlated with the nodal status. Although CTCs and DTCs were significantly associated with the presence of distant metastasis, no statistically significant association was found with the overall survival and the time of relapse or death, probably due to the small sample size. While no significant correlation was found between the detection of CTCs and DTCs, their combination, however, showed a significant association with recurrence-free survival time. The results of this study showed the potential of CTCs and DTCs for predicting local relapse with higher sensitivity than routine staging procedures. One year later, Inhestern et al. [43] analyzed the prognostic relevance of CTCs in 40 patients with advanced nonmetastatic oral (38%) and oropharyngeal (63%) cancer treated by induction therapy before surgery and postoperative radiotherapy. Samples were taken before each cycle of induction chemotherapy, before surgery, before postoperative radiotherapy, and at the end of treatment. At baseline, CTCs were found in 80% of patients and no CTCs at the end of therapy were found in 3% of patients. At baseline, higher CTC levels were associated with higher risk of recurrence during therapy. In addition, patients with high CTC levels during the complete course of treatment had a significantly lower overall survival. They also found postsurgical increased levels of CTCs (that decreased after postoperative radiotherapy), but they were not associated with a negative prognostic impact. The results of this study established CTCs as an independent prognostic marker when OSCC is treated by docetaxel-, cisplatin- and fluorouracil-induction chemotherapy, surgery, and postoperative (chemo) radiation. In addition to direct methods to quantify CTCs such as CellSearch, several authors used indirect strategies to quantify CTC levels. Toyoshima et al. [48] analyzed after surgery the presence of several cytokeratin (CK) mRNA transcripts (*CK 17*, *CK 18*, *CK 19*, and *CK 20*) in peripheral blood to determine their value for detecting CTCs. Although the other CKs were detected in very few patients, CK20 was found in 35.0%. Besides, detection of CK20 was significantly higher in late stages and was associated with poor disease-free survival. On the other hand, in metastatic patients, several studies have also shown the prognostic value of CTC counts. For instance, Grisanti et al. [35] found that CTCs were detected in one out of three patients with recurrent or metastatic head and neck carcinomas. The univariate analysis indicated that patients with 0–1 CTCs had a better prognosis than those with ≥2 CTCs, and in multivariate analysis, ≥1 CTC was associated with a poor prognosis in terms of progression-free survival.

Importantly, not only single CTCs, but also CTC clusters (≥3 tumour cells) or circulating tumour microemboli have been reported [29] as prognostic factors. It is important to bear in mind that CTC cluster count is normally underestimated due to their shorter circulation half-life, showing a faster entrapment in distant organs where they can start new tumour locations [29]. Kulasinghe et al. [51] evaluated by spiral microfluidic technology the CTC levels in 60 treatment-naive head and neck cancer patients (40% in the oral cavity), including early and late tumour stages. Single CTCs appeared in 33.3% of patients (ranging from 1–10 CTCs/5 mL) with stages I–IV, and CTC clusters were detected in 25% of patients (ranging from 1–3 clusters/5 mL, including 3–16 cells) with stage IV. A total of seven stage IV patients developed lung/liver metastasis during the follow-up, of which six patients showed CTC clusters. Furthermore, some of the clusters showed the incorporation of white blood cells, which could facilitate the clusters’ ability to evade immune attacks.

In addition to the CTC count, different studies have attempted the molecular characterization of CTCs in oral tumours. In these studies, CTCs were characterized by both epithelial and mesenchymal markers [30,45,47,48]. Grisanti et al. [35] observed in patients with locally advanced and recurrent metastatic head and neck cancer (32% of the oral cavity) that EGFR expression could be modulated over time, with the presence of EGFR at any time point being low (45%) when compared with the primary tumour, which could identify cancer cells that were more likely to metastasize. Another study demonstrated the value of the combined analysis of podoplanin (a transmembrane protein with several functions in lymphatic vessel formation and cellular cytoskeleton remodeling) and EpCAM to prognostic tumour evolution in locally advanced and metastatic head and neck cancer, where primary tumour sites were mainly in the oral cavity. In this study, the ratio of podoplanin-positive/EpCAM-positive CTCs showed an independent prognostic value with respect to the type of treatment and tumour staging [49]. To provide more knowledge regarding CTC biology for OSCC, Oliveira-Costa et al. [47] analyzed the gene expression profile of OSCC tumours to identify biomarkers that increased or decreased during tumour progression (through T1 to T4 stages). A total of 879 transcripts were significantly increased or decreased from the T1 to T4 stage, of which six (PD-L1, HOXB9, DHDH, BLNK, ZNF813, and IL6ST) were validated by qRT-PCR in tissue samples. The expression of four of these markers was evaluated in CTCs, observing an increase in PD-L1, HOXB9, and ZNF813 and an important decrease in BLNK expression. Importantly, their expression pattern was in concordance with the findings in the primary tumours. Due to PD-L1 inducing an exhaustion state in T cells and reducing the capability of a T-cell-mediated response, the study hypothesized that OSCC patients could benefit from anti-PD-L1 therapy, which could probably be monitored by identifying individuals with PD-L1+ CTCs. The comparison of metastasis-associated genes between the primary tumour and CTCs was also analyzed in other studies. For example, Ito et al. studied this correlation in 22 OSCC patients. CK9 expression was detected in 80% of the samples from primary tumours and 90% from CTCs. In primary tumours, CD44s expression was detected in 20% of the cases, while CD44v6 and v9 expression was detected in 30%. However, CTCs were CD44s-positive in 90% of the samples and CD44v6- and v9-positive in a total of 50%. Importantly, MMPs (MMP-1, -2, -7, -9) expression was detected with a range from 20% to 60% in tissue, although most of the CTCs did not express any MMPs. These results confirmed the higher expression of metastasis-associated genes in CTCs, facilitating the development of metastasis [45].

Overall, nowadays there are a great number of technologies for CTCs analyses. However, these analyzes are not currently being used to manage patients’ treatment and monitoring. The key point for the future implementation of CTCs at clinical levels is related to the sensibility and the versatility of these techniques. This means that we need a very sensitive system to detect CTCs in all metastatic patients and also in a high percentage of early stages, which can enrich a wide variety of CTCs using both epithelial and mesenchymal markers. Furthermore, regarding CTC capture, single-cell sequencing needs improvement. The development of more advanced sequencing technologies and also new bioinformatic tools are necessary to obtain a better comprehension of these cells’ biology and the improved use of this information to guide treatment selection and monitoring.

### 2.2. Circulating Cell-Free DNA (cfDNA)

cfDNA originates from apoptotic or necrotic cells that release it into the bloodstream and other biofluids by all type of cells including both nonmalignant host cells and tumour cells [52,53]. ctDNA can be differentiated from cfDNA on the basis of somatic mutations. ctDNA represents a small variable population within a large population of cfDNA. cfDNA is highly fragmented, differentiating short (70–200 bp) or long (up to 21 kb) fragments of double-stranded DNA. It can be found in blood, saliva, plasma, urine, cerebrospinal fluid, and other bodily fluids [52,54,55,56]. Numerous tumour characteristics, such as overall size, staging, vascularity, cellular turnover, location, and response to treatment, were shown to be associated with ctDNA concentrations [57]. Nowadays, cfDNA is being studied extensively since it is easy to analyze compared with other biomarkers [58] and contains specific tumour- and metastasis-related alterations, such as single-nucleotide mutations, methylation changes, and copy-number variations [52]. Importantly, it has been shown that ctDNA represents the tumour genome from primary tumours and metastasis and reflects the clonality of tumour cells [59,60].

Different technologies for ctDNA detection have been developed during the last years, allowing it to be analyzed from the level of a point mutation to that of the entire genome [57]. Classical methods of analyzing ctDNA include quantitative real-time PCR, fluorescent assays, and spectrophotometric strategies. Digital PCR-based technologies are highly sensitive techniques designed for the detection of specific point mutations, copy-number variations, short indels, and gene fusions. These technologies include droplet-PCR, microfluidic systems for parallel PCR, and BEAMing (beads, emulsions, amplification, and magnetics). Next-generation sequencing technologies are the other alternative for cfDNA characterization. These technologies allow high-throughput and relatively low-cost analyses to identify ctDNA alterations across wide genomic regions and have the advantage of not requiring prior knowledge of the genetic alterations of the tumour [18].

To date, several studies have applied cfDNA analyses in OSCCs. Shukla et al. [61] analyzed by spectrophotometry the quantity of DNA in the plasma of 390 patients (90 potentially malignant lesions, 150 OSCCs, and 150 post-treatment OSCCs) and 150 healthy controls, but no significant differences were observed between the groups. One possible reason for this finding is that thanks to the rich lymphatic drainage of the oral mucosa, cfDNA is prevented from entering the bloodstream. More recently, Perdomo et al. [62] reported the detection of ctDNA mutations in head and neck cancers, of which 41 were oral cavity cancers, using two different approaches. In the first approach, the presence of ctDNA mutations in five genes (*TP53*, *NOTCH1*, *CDKN2A*, *CASP8* and *PTEN*), previously identified in tumour samples, were examined in plasma, finding a total of 18 mutations in 42% of the patients, including those in the early stages. In the second approach, they analyzed TP53 mutation by sequencing tumour tissue, plasma, and oral rinses, showing *TP53* mutations in 36%, 3%, and 26% of patients, respectively. Importantly, concordance of mutation detection was low between tumour tissue, oral rinses (11%), and plasma (2.7%).

HPV detection using cfDNA could also be of great value for OSCC. Mazurek et al. [63] analyzed the cfDNA levels of 200 head and neck squamous cell carcinomas by examining HPV16/18, KRAS, and EGFR mutations via q-PCR. A higher level of the total cfDNA was found in patients with oropharyngeal squamous cell carcinoma in comparison with other head and neck squamous cell carcinomas. The level of cfDNA increased in patients with both greater lymph node affectation and tumoural stage. From all patients, 14% showed positivity for HPV, most of whom were HPV16-positive (96.4%), while somatic EGFR and KRAS mutations were not detected. These results showed that HPV cfDNA tests could be used for the early detection and monitoring of HPV-positive head and neck squamous cell carcinomas. A total of 30 patients out of 93 were diagnosed as HPV16-positive in their primary head and neck tumours, but none showed HPV18. The anatomic predilection was for oropharynx, with 29 patients showing HPV16. Interestingly, when HPV detection was compared in plasma with respect to saliva, results showed that HPV was found in 86% of plasma samples versus 40% of salivary samples. Digital PCR was used to quantify HPV in both fluids [64].

Microsatellite instability analyses using cfDNA have also shown promise for the prognosis and the monitoring of OSCC. Hamana et al. [65] analyzed nine microsatellite markers (D5s178, D9S104, IFNA, D11S910, D11S1356, D13S273, TP53, D18S46 and D22S274) in the tissue and serum of OSCC patients at three different time points (preoperatively, postoperatively, and four weeks after surgery). In this study, it was shown that the cfDNA reflected the characteristics of the tumour, since allelic imbalance patterns in serum were associated with the presence of allelic imbalance in paired tumour tissue. Serum allelic imbalances were observed both in preoperative (44%) and postoperative (20%) samples. Among the patients, those with an allelic imbalance four weeks postsurgery developed metastasis, whereas those without evidence of an allelic imbalance had no recurrence of the disease. Interestingly, IFNA at the 9p21 locus was the most frequently altered chromosome, found in 40% of patients with some allelic imbalance in plasma. In another study, Kakimoto et al. [66] analyzed a panel of nine microsatellite markers (D2S1327, D2S206, D3S1007, D3S1079, D3S966, D21S36, D21S11, D21S1254 and D21S369) in blood and tumour tissue samples before and one month after surgery. The presence of an allelic imbalance in tumour DNA was observed in the serum of 90% of patients. Further, the detection of allelic imbalance in serum postoperatively was associated with a poor prognosis. Based on these results, microsatellite analysis could help to assess risk of recurrence, metastasis, and death in OSCC. Similarly, Nunes et al. [67] analyzed eight microsatellite markers (D2S123, D13S308E, D5S1501, D1S3721, D12S1052, D17S974, D17S1294 and D13S800) in 91 stage I to IV head and neck tumours (51% from oral origin). Fifty-eight patients presented microsatellite alterations in the tumour, mainly at D17S974 and D13S800. Of these, 17 patients showed the same profile in plasma. Furthermore, the detection of ctDNA was independent of the stage of the tumour, indicating its utility for early diagnosis or screening. Regarding the association between ctDNA levels and tumour burden, a recent preclinical study using mice models of head and neck cancer found a correlation between ctDNA levels and tumour burden after monitoring primary tumour and metastatic lymph node volume by CT scan. Importantly, they detected ctDNA changes before a tumour could be spotted by CT scan [68].

Finally, aberrant DNA methylation patterns can be assessed accurately in cfDNA [69]. SHOX2 and SPEPT9 are the methylation markers with the highest level of validation in biofluids of different cancers remote from the oral cavity [70,71]. Both biomarkers have been validated in a large prospective cohort of 649 head and neck cancer patients as valuable biomarkers for tumour diagnosis, showing 59% of patients as methylation-positive at 96% specificity. Methylation levels also correlated with survival rates and showed value in monitoring treatment efficiency [72].

Although evidence exists regarding the value of ctDNA in different clinical scenarios, there are still some issues to solve before reaching general use at hospitals. Firstly, ctDNA is detected in a very low ratio in early stages, being undetectable without highly sensitive techniques [42,73]. Secondly, due to tumour heterogeneity and evolution, multiplexed assays are need to analyze several mutations simultaneously and to solve the limitation of the low levels of cfDNA present in some patients [73]. Finally, several methods have been developed to detect ctDNA, but there is still a lack of a standardized method, which is essential for its clinical application together with the need of reducing the analysis cost.

### 2.3. Exosomes

An additional approach for liquid biopsy analyses involves bioactive vesicles described as exosomes by Pan and Johnstone in 1983 [74]. Exosomes are small membrane vesicles with diameters ranging from 40–150 nm and a lipid bilayer membrane [75]. Exosomes present an enriched surface of proteins such as fusion and transport proteins (Rab GTPases, annexins, and flotillin), components of the endosomal sorting complexes required for transport complexes (ESCRT complexes), heat shock proteins (HSP70, HSP90), integrins, and tetraspanins (CD9, CD63, CD81, CD82) [75,76]. Research has demonstrated the presence of exosomes in the tumour microenvironment, suggesting its importance in tumourigenesis, tumour invasion, and metastasis, since they can act as promoters of tumour progression or possess an antitumour function [75,77]. Depending on the mode of biogenesis, cell type, and physiological conditions, exosomes contain several types of parent cell-derived bioactive molecules, such as proteins, lipids, mRNAs, microRNAs (miRNAs), long noncoding RNAs (lncRNAs), genomic DNA, cDNA, and mitochondrial DNA (mtDNA) [78]. Exosomes can be abundantly released by different types of cells into numerous biological fluids such as urine, semen, saliva, amniotic fluid, cerebrospinal fluid, lymph, bile, ascites, tears, breast milk, and blood, both in healthy and diseased conditions [79,80,81].

In OSCC, exosomes have shown to be key components in the tumour microenvironment, increasing the transforming growth factor-β (TGF-β) signaling pathway, which contributes to progression and drug resistance of OSCC [82]. Exosomes secreted by tumour cells have been involved in tumour angiogenesis and metastasis under hypoxic conditions [83,84]. Li et al. [84] found that exosomes derived from hypoxic OSCC cells increased the migration and invasion of OSCC cells in HIF1α- and HIF-2α-dependent ways. A miRNA expression profile of hypoxic tumour-derived exosomes showed higher levels of miR-21, miR-205, and miR-148b compared to exosomes with a normal oxygen concentration. According to their results, expression levels of exosomal miR-21 were associated with cell motility and invasion in hypoxic conditions. In addition, in OSCC patients, a correlation was found between circulating exosomal miR-21 levels and metastasis in the lymph nodes.

Recent data also suggest that tumour exosomes play an important role in immune suppression, enhancing tumour development and progression. In fact, tumour exosomes can communicate with immune cells through immunoinhibitory (protumour) and immunostimulatory (antitumour) signals in the tumour microenvironment [85]. Theodoraki et al. [86] evaluated the clinical significance of PD-L1+ exosomes in the plasma of head and neck cancer patients, due to the important role of the PD-1/PD-L1 pathway in immune regulation. A significant positive correlation was found between high-PD-L1 cargo of circulating exosomes and advancing disease, high-tumour stages, and positive lymph nodes. Nevertheless, plasma levels of soluble PD-L1 (sPD-L1) did not correlate with clinicopathological parameters, and there was no correlation between the frequency of PD-L1+ exosomes and levels of sPD-L1. Interestingly, this experiment showed that high PD-L1 exosomes downregulated CD69 expression on effector T cells, whereas the presence of anti-PD-1 antibody reduced downregulation of CD69 expression levels. These findings revealed the potential of circulating PD-L1+ exosomes to modify immune responses and indirectly influence disease activity. Ludwig et al. [87] showed that the plasma-derived exosomes of cancer patients (active disease) induced significantly stronger apoptosis of CD8^+^ T cells, suppression of T-cell proliferation, downregulated NKG2D expression levels in natural killer cells, and upregulation of regulatory T-cell (Treg) suppressor functions. Furthermore, exosomes could be used to discriminate between active-disease cancer patients and those with no evident disease after oncologic therapies. Their results showed that exosome-induced immune suppression is correlated with disease activity, suggesting the potential of plasma exosomes as biomarkers of the progression of head and neck cancer.

Oral fluid-derived exosomes have been characterized morphologically in oral cancer by Zlotogorski-Hurvitz et al. [88]. They analyzed the expression of three exosomal markers (CD9, CD81, and CD63), showing a mean concentration higher for CD63 and lower for CD9 and CD81 between cancer patients and healthy individuals, although significant statistical differences were observed only for CD81 levels. Further, a nanoparticle tracking analysis revealed a significantly higher concentration of particles in oral cancer compared to healthy individuals. Also, the average modal size of the nanoparticles in oral cancer samples was significantly larger compared to the control health samples. These findings, based on the molecular and morphological characteristics of exosomes, suggest their potential as specific biomarkers for oral cancer detection. Recently, Rabinowitts et al. [89] performed a comparative analyses of microRNAs expression among benign and malignant tissue and plasma of patients with tongue cancer. Of a total of 359 miRNAs, 16 were differentially expressed between tumour and matched benign tissue. Of them, nine were upregulated (miR-19a, miR-512-3p, miR-27b, miR-20a, miR-28-3p, miR-200c, miR-151-3p, hsa-miR-223, miR-20b), whereas seven were downregulated (miR-22, miR-516-3p, miR-370, miR-139-5p, let-7e, miR-145-3p, miR-30c) in tumour tissue compared to matched benign tissue. Importantly, all upregulated and six downregulated miRNAs were identified in circulating exosomes, excluding miR-516-3p, which was only in tumour tissue. The discovery of this miRNA signature in both the tumours and plasma of patients with tongue squamous cell carcinoma highlights the importance of miRNAs (both free and within exosomes) as potential biomarkers for the diagnosis of tongue cancer. Additionally, circulating miRNAs packaged in protein complexes or encapsulated in microvesicles are protected against the activity of the RNAses present in blood, representing a more reliable method for evaluating circulating tumour-miRNA signatures [90].

Regarding the challenges for the clinical implementation of circulating exosomes as a clinical biomarker, several questions need to be resolved. Although in vitro or in vivo xenograft models have investigated the function of tumour exosomes, this field requires the design of exhaustive studies focusing on the mechanisms of biogenesis, cargo sorting, or the physiological relevance of the exosome [76,81]. Therefore, the heterogeneity of tumour exosomes and its functional significance should be explored with in vivo studies. In addition, a consensus on the isolation strategy, classification, and contents of exosomes must be clarified to generate the common and standardized protocols required for clinical use.

### 2.4. Salivary Biomarkers for Oral Cancer

The use of salivary biomarkers has gained increased attention as a novel, noninvasive method for cancer diagnosis. Currently, multiple salivary molecules can be used as biomarkers in cancer for diagnosis, prognosis, treatment monitoring, and pharmacogenetic studies [91,92]. Keeping in mind that saliva can be considered as “the mirror of the body” [93], it has become an attractive clinical tool due to its easy collection and simple storage [94]. Five diagnostic alphabets have already been characterized in salivary samples: proteome, transcriptome, micro-RNAs, metabolome, and microbiome [95]. Only a few studies analyze saliva to find biomarkers for oral cancer, although the results are promising. In a study performed by Spafford et al. [96], a total of 44 head and neck squamous cell carcinoma patients (13 of which were located in the oral cavity) and 43 healthy subjects were analyzed to find tumour-specific microsatellite alterations in the DNA from exfoliated salivary oral cells. A panel of 23 microsatellite markers were studied (D9S753, D20S77, UT5307, D9S242, CSFIR-6, D11S488, ACTBP2, D8S321, UT5320, D9S171, D9S162, D20S82, D20S85, Li7686, FGA, D9SIFNA, D11S654, D3S1560, D3S1286, D3S1289, D17S695, D17S654, and D17S656). Loss of heterozygosity or microsatellite instability of at least of one marker was found in 86% (38/44) of primary tumours and none of them were found in the healthy control group. Importantly, identical alterations were found in saliva from 35 of these 38 cases (92%). In another study using tissue and saliva from 23 oral and pharyngeal cancer patients, a panel of eight microsatellite markers (D3S1289, D3S1300, D8S320, D8S321, D9S242, D11S488, and D20S82) was chosen because they show a high incidence of microsatellite abnormalities in head and neck cancers. Microsatellite instability was found in 22% of the tumour samples; of these, 80% had microsatellite instability in salivary samples also [97].

Mitochondrial DNA (mtDNA) has also been studied in saliva samples from head and neck cancer patients [98,99]. Jiang et al. [99] showed that mtDNA content (Cox I and Cox II) in the saliva of head and neck cancer patients was elevated in comparison with the healthy control group. Moreover, a salivary mtDNA was associated with the tumoural stage. Later on, the same researchers analysed saliva from 76 patients with head and neck cancer (oral cavity cancer in 54.7% of these cases) and described a reduction of mtDNA content in both mitochondrial subunit genes (cytochrome c oxidase I, Cox I; and cytochrome c oxidase II, Cox II) in comparison to pretreatment saliva. Further, a multivariate analysis indicated that postoperative radiation and smoking were influencing factors for mtDNA content.

On the other hand, several oxidative stress-related parameters and antioxidant salivary profiles were analyzed in 25 OSCC patients and 25 healthy controls [100]. Salivary DNA and proteins were found to be highly oxidized and all salivary nitrosamines were significantly increased, while salivary antioxidants were decreased. These findings could explain the oxidative damage to the DNA and proteins, showing a link between free radicals and antioxidants in saliva with OSCC. In addition, an electrophoretic technique with ethidium bromide has been used to characterize salivary DNA in a sample of 67 OSCC patients versus 27 healthy individuals. Sensitivity and specificity values of 88.9% and 94.0%, were found from the fluorescence emission spectra, and 88.9% and 92.5%, with fluorescence excitation [101].

Salivary-tumour-derived exosomes represent a potential alternative for the identification of novel biomarkers for oral cancer [102]. Sharma et al. [103] evaluated the exosome morphology and expression of the biomolecular surface receptor CD63 in salivary samples of oral cancer patients and healthy controls. Based on their results, oral-cancer-derived exosomes presented a variable size (20–400 nm), a higher intervesicular aggregation, and possess a higher expression of CD63 molecules compared to normal saliva exosomes. Recently, Langevin et al. [104] characterized the exosomal miRNA secretome of head and neck squamous cell carcinoma and nonpathologic oral epithelial cells through miRNA sequencing. Although the different head and neck squamous cell carcinoma cell lines showed a high degree of overlap of differentially secreted exosomal miRNAs relative to control cells, a total of 22 miRNA transcripts were detected only in cancer exosomes. Of them, eight candidate exosomal miRNAs were assessed in human saliva samples, finding increased levels of miR-486-5p, miR-486-3p, and miR10b-5p in the saliva of head and neck squamous cell carcinoma patients when compared with healthy individuals. Thus, they demonstrated that salivary exosomal miRNAs could serve as noninvasive diagnostic biomarkers for oral cancer. In addition, a wide variety of circulating free miRNAs that have been differentially expressed in the saliva of OSCC patients may be potential novel tumour markers for the diagnosis and monitoring of cancer [105,106].

## 3. Future Perspectives of Liquid Biopsy

In recent years, liquid biopsy has emerged as a potential noninvasive approach in precision, personalized medicine. The detection and analysis of CTCs, ctDNA, and circulating exosomes represent a promising opportunity for early cancer detection, molecular profiling analysis, monitoring of treatment response, and detection of minimal residual disease and relapse. Compared to other cancer anatomic locations, in oral cancer, the impact of liquid biopsies in the clinical setting is still limited, requiring further research for effective implementation. The discovery of a robust panel of sensitive and specific circulating biomarkers for oral cancer at different stages would help clinicians improve diagnosis and prognosis, and mark the beginning of personalized medicine. A better knowledge of the biology and origin of circulating biomarkers would be the key for the development of effective therapies for and the management of oral cancer. Liquid biopsy for oral cancer is in its infancy, so research efforts should be addressed to perform large, prospective multicenter studies that investigate the role of CTCs, ctDNA, and exosomes in oral cancer.

## References

[1] Globocan. http://globocan.iarc.fr/Default.aspx.

[2] Ferlay J., Soerjomataram I.I., Dikshit R., Eser S., Mathers C., Rebelo M., Parkin D.M., Forman D.D., Bray F. (2015). Cancer incidence and mortality worldwide: Sources, methods and major patterns in GLOBOCAN 2012. Int. J. Cancer.

[3] Huang Z., Huang D., Ni S., Peng Z., Sheng W., Du X. (2010). Plasma microRNAs are promising novel biomarkers for early detection of colorectal cancer. Int. J. Cancer.

[4] National Cancer Institute (US). https://www.ncbi.nlm.nih.gov/books/NBK66005/.

[5] Beynon R.A., Lang S., Schimansky S., Penfold C.M., Waylen A., Thomas S.J., Pawlita M., Waterboer T., Martin R.M., May M. (2018). Tobacco smoking and alcohol drinking at diagnosis of head and neck cancer and all-cause mortality: Results from head and neck 5000, a prospective observational cohort of people with head and neck cancer. Int. J. Cancer.

[6] Yete S., D’Souza W., Saranath D. (2018). High-risk human papillomavirus in oral cancer: Clinical implications. Oncology.

[7] Brasil V.L.M., Ramos Pinto M.B., Bonan R.F., Kowalski L.P., da Cruz Pérez D.E. (2018). Pesticides as risk factors for head and neck cancer: A review. J. Oral Pathol. Med..

[8] Speight P.M., Khurram S.A., Kujan O. (2017). Oral potentially malignant disorders: Risk of progression to malignancy. Oral Surg. Oral Med. Oral Pathol. Oral Radiol..

[9] Katsanos K.H., Roda G., Brygo A., Delaporte E., Colombel J.F. (2015). Oral cancer and oral precancerous lesions in inflammatory bowel diseases: A Systematic Review. J. Crohn’s Colitis.

[10] Warnakulasuriya S. (2009). Global epidemiology of oral and oropharyngeal cancer. Oral Oncol..

[11] Sinevici N., O’sullivan J. (2016). Oral cancer: Deregulated molecular events and their use as biomarkers. Oral Oncol..

[12] Krishna A., Singh S., Kumar V., Pal U. (2015). Molecular concept in human oral cancer. Natl. J. Maxillofac. Surg..

[13] Ramos-García P., Gil-Montoya J.A., Scully C., Ayén A., González-Ruiz L., Navarro-Triviño F.J., González-Moles M.A. (2017). An update on the implications of cyclin D1 in oral carcinogenesis. Oral Dis..

[14] Dzebo S., Mahmutovic J., Erkocevic H., Foco F. (2017). Frequency of depression and its correlation with quality of life of patients with oral cavity cancer. Mater. Socio Med..

[15] Manasa V.G., Kannan S. (2017). Impact of microRNA dynamics on cancer hallmarks: An oral cancer scenario. Tumor Biol..

[16] Bellairs J.A., Hasina R., Agrawal N. (2017). Tumor DNA: An emerging biomarker in head and neck cancer. Cancer Metastasis Rev..

[17] Rübben A., Araujo A. (2017). Cancer heterogeneity: Converting a limitation into a source of biologic information. J. Transl. Med..

[18] Siravegna G., Marsoni S., Siena S., Bardelli A. (2017). Integrating liquid biopsies into the management of cancer. Nat. Rev. Clin. Oncol..

[19] De Sá Junior P.L., Câmara D.A.D., Porcacchia A.S., Fonseca P.M.M., Jorge S.D., Araldi R.P., Ferreira A.K. (2017). The roles of ROS in cancer heterogeneity and therapy. Oxid. Med. Cell. Longev..

[20] Wang J., Chang S., Li G., Sun Y. (2017). Application of liquid biopsy in precision medicine: Opportunities and challenges. Front. Med..

[21] Jia S., Zhang R., Li Z., Li J. (2017). Clinical and biological significance of circulating tumor cells, circulating tumor DNA, and exosomes as biomarkers in colorectal cancer. Oncotarget.

[22] Ma M., Zhu H., Zhang C., Sun X., Gao X., Chen G. (2015). “Liquid biopsy”-ctDNA detection with great potential and challenges. Ann. Transl. Med..

[23] Peng M., Chen C., Hulbert A., Brock M.V., Yu F. (2017). Non-blood circulating tumor DNA detection in cancer. Oncotarget.

[24] Di Meo A., Bartlett J., Cheng Y., Pasic M.D., Yousef G.M. (2017). Liquid Biopsy: A step forward towards precision medicine in urologic malignancies. Mol. Cancer.

[25] Sumanasuriya S., Lambros M.B., de Bono J.S. (2017). Application of liquid biopsies in cancer targeted therapy. Clin. Pharmacol. Ther..

[26] Huang S.H., O’Sullivan B. (2017). Overview of the 8th edition TNM classification for head and neck cancer. Curr. Treat. Options Oncol..

[27] Xu L., Li J.H., Ye J.M., Duan X.N., Cheng Y.J., Xin L., Liu Q., Zhou B., Liu Y.H. (2017). A retrospective survival analysis of anatomic and prognostic stage group based on the american joint committee on cancer 8th edition cancer staging manual in luminal B human epidermal growth factor receptor 2-negative breast cancer. Chin. Med. J. (Engl.).

[28] Singh M., Yelle N., Venugopal C., Singh S.K. (2018). EMT: Mechanisms and therapeutic implications. Pharmacol. Ther..

[29] Au S.H., Edd J., Stoddard A.E., Wong K.H.K., Fachin F., Maheswaran S., Haber D.A., Stott S.L., Kapur R., Toner M. (2017). Microfluidic isolation of circulating tumor cell clusters by size and asymmetry. Sci. Rep..

[30] Patel S., Shah K., Mirza S., Shah K., Rawal R. (2016). Circulating tumor stem like cells in oral squamous cell carcinoma: An unresolved paradox. Oral Oncol..

[31] Aceto N., Bardia A., Miyamoto D.T., Donaldson M.C., Wittner B.S., Spencer J.A., Yu M., Pely A., Engstrom A., Zhu H. (2014). Circulating tumor cell clusters are oligoclonal precursors of breast cancer metastasis. Cell.

[32] Anitha N., Jimson S., Masthan K.M.K., Jacobina J.J. (2015). Circulating tumor cells in oral squamous cell carcinoma—An enigma or reality?. J. Pharm. Bioallied Sci..

[33] Garg M. (2018). Epithelial plasticity and metastatic cascade. Expert Opin. Ther. Targets.

[34] Lowes L.E., Allan A.L. (2018). Circulating tumor cells and implications of the epithelial-to-mesenchymal transition. Adv. Clin. Chem..

[35] Grisanti S., Almici C., Consoli F., Buglione M., Verardi R., Bolzoni-Villaret A., Bianchetti A., Ciccarese C., Mangoni M., Ferrari L. (2014). Circulating tumor cells in patients with recurrent or metastatic head and neck carcinoma: Prognostic and predictive significance. PLoS ONE.

[36] Krebs M.G., Metcalf R.L., Carter L., Brady G., Blackhall F.H., Dive C. (2014). Molecular analysis of circulating tumour cells—Biology and biomarkers. Nat. Rev. Clin. Oncol..

[37] Yu M., Stott S., Toner M., Maheswaran S., Haber D.A. (2011). Circulating tumor cells: Approaches to isolation and characterization. J. Cell Biol..

[38] Zhou L., Dicker D.T., Matthew E., El-Deiry W.S., Alpaugh R.K. (2017). Circulating tumor cells: Silent predictors of metastasis. F1000Reserch.

[39] Buglione M., Grisanti S., Almici C., Mangoni M., Polli C., Consoli F., Verardi R., Costa L., Paiar F., Pasinetti N. (2012). Circulating tumour cells in locally advanced head and neck cancer: Preliminary report about their possible role in predicting response to non-surgical treatment and survival. Eur. J. Cancer.

[40] Chen J.Y., Chang Y.C. (2017). Strategies for isolation and molecular profiling of circulating tumor cells. Adv. Exp. Med. Biol..

[41] Harouaka R., Kang Z., Zheng S.Y., Cao L. (2014). Circulating tumor cells: Advances in isolation and analysis, and challenges for clinical applications. Pharmacol. Ther..

[42] Ignatiadis M., Lee M., Jeffrey S.S. (2015). Circulating tumor cells and circulating tumor DNA: Challenges and opportunities on the path to clinical utility. Clin. Cancer Res..

[43] Inhestern J., Oertel K., Stemmann V., Schmalenberg H., Dietz A., Rotter N., Veit J., Görner M., Sudhoff H., Junghanb C. (2015). Prognostic role of circulating tumor cells during induction chemotherapy followed by curative surgery combined with postoperative radiotherapy in patients with locally advanced oral and oropharyngeal squamous cell cancer. PLoS ONE.

[44] Gröbe A., Blessmann M., Hanken H., Friedrich R.E., Schön G., Wikner J., Effenberger K.E., Kluwe L., Heiland M., Pantel K. (2014). Prognostic relevance of circulating tumor cells in blood and disseminated tumor cells in bone marrow of patients with squamous cell carcinoma of the oral cavity. Clin. Cancer Res..

[45] Ito H., Hatori M., Kinugasa Y., Irie T., Tachikawa T., Nagumo M. (2003). Comparison of the expression profile of metastasis-associated genes between primary and circulating cancer cells in oral squamous cell carcinoma. Anticancer Res..

[46] Kawamata H., Uchida D., Nakashiro K., Hino S., Omotehara F., Yoshida H., Sato M. (1999). Haematogenous cytokeratin 20 mRNA as a predictive marker for recurrence in oral cancer patients. Br. J. Cancer.

[47] Oliveira-Costa J.P., Carvalho A.F., Silveira G.G., Amaya P., Wu Y., Park K.J., Gigliola M.P., Lustberg M., Buim M.E., Ferreira E.N. (2015). Gene expression patterns through oral squamous cell carcinoma development: PD-L1 expression in primary tumor and circulating tumor cells. Oncotarget.

[48] Toyoshima T., Vairaktaris E., Nkenke E., Schlegel K.A., Neukam F.W., Ries J. (2009). Hematogenous Cytokeratin 20 mRNA detection has prognostic impact in oral squamous cell carcinoma: Preliminary results. Anticancer Res..

[49] Hsieh J.C.H., Lin H.C., Huang C.Y., Hsu H.L., Wu T.M.H., Lee C.L., Chen M.C., Wang H.M., Tseng C.P. (2015). Prognostic value of circulating tumor cells with podoplanin expression in patients with locally advanced or metastatic head and neck squamous cell carcinoma. Head Neck.

[50] Partridge M., Brakenhoff R., Phillips E., Ali K., Francis R., Hooper R., Lavery K., Brown A., Langdon J. (2003). Detection of rare disseminated tumor cells identifies head and neck cancer patients at risk of treatment failure. Clin. Cancer Res..

[51] Kulasinghe A., Schmidt H., Perry C., Whitfield B., Kenny L., Nelson C., Warkiani M.E., Punyadeera C. (2018). A collective route to head and neck cancer metastasis. Sci. Rep..

[52] Salvi S., Gurioli G., De Giorgi U., Conteduca V., Tedaldi G., Calistri D., Casadio V. (2016). Cell-free DNA as a diagnostic marker for cancer: Current insights. OncoTargets Ther..

[53] Van Ginkel J.H., Slieker F.J.B., de Bree R., van Es R.J.J., Willems S.M. (2017). Cell-free nucleic acids in body fluids as biomarkers for the prediction and early detection of recurrent head and neck cancer: A systematic review of the literature. Oral Oncol..

[54] Nishita D.M., Jack L.M., McElroy M., McClure J.B., Richards J., Swan G.E., Bergen A.W. (2009). Clinical trial participant characteristics and saliva and DNA metrics. BMC Med. Res. Methodol..

[55] Fliss M.S., Usadel H., Caballero O.L., Wu L., Buta M.R., Eleff S.M., Jen J., Sidransky D. (2000). Facile detection of mitochondrial DNA mutations in tumors and bodily fluids. Science.

[56] Offin M., Chabon J.J., Razavi P., Isbell J.M., Rudin C.M., Diehn M., Li B.T. (2017). Capturing genomic evolution of lung cancers through liquid biopsy for circulating tumor DNA. J. Oncol..

[57] Chang Y., Tolani B., Nie X., Zhi X., Hu M., He B. (2017). Review of the clinical applications and technological advances of circulating tumor DNA in cancer monitoring. Ther. Clin. Risk Manag..

[58] Aro K., Wei F., Wong D.T., Tu M. (2017). Saliva liquid biopsy for point-of-care applications. Front. Public Health.

[59] Murtaza M., Dawson S.J., Tsui D.W.Y., Gale D., Forshew T., Piskorz A.M., Parkinson C., Chin S.F., Kingsbury Z., Wong A.S.C. (2013). Non-invasive analysis of acquired resistance to cancer therapy by sequencing of plasma DNA. Nature.

[60] Alix-Panabières C., Pantel K. (2013). Real-time liquid biopsy: Circulating tumor cells versus circulating tumor DNA. Ann. Transl. Med..

[61] Shukla D., Kale A.D., Hallikerimath S., Yerramalla V., Subbiah V. (2013). Can quantifying free-circulating DNA be a diagnostic and prognostic marker in oral epithelial dysplasia and oral squamous cell carcinoma?. J. Oral Maxillofac. Surg..

[62] Perdomo S., Avogbe P.H., Foll M., Abedi-Ardekani B., Lescher Facciolla V., Anantharaman D., Chopard P., Le Calvez-Kelm F., Vilensky M., Polesel J. (2017). Circulating tumor DNA detection in head and neck cancer: Evaluation of two different detection approaches. Oncotarget.

[63] Mazurek A.M., Rutkowski T., Fiszer-Kierzkowska A., Małusecka E., Składowski K. (2016). Assessment of the total cfDNA and HPV16/18 detection in plasma samples of head and neck squamous cell carcinoma patients. Oral Oncol..

[64] Wang Y., Springer S., Mulvey C.L., Silliman N., Schaefer J., Sausen M., James N., Rettig E.M., Guo T., Pickering C.R. (2015). Detection of somatic mutations and HPV in the saliva and plasma of patients with head and neck squamous cell carcinomas. Sci. Transl. Med..

[65] Hamana K., Uzawa K., Ogawara K., Shiiba M., Bukawa H., Yokoe H., Tanzawa H. (2005). Monitoring of circulating tumour-associated DNA as a prognostic tool for oral squamous cell carcinoma. Br. J. Cancer.

[66] Kakimoto Y., Yamamoto N., Shibahara T. (2008). Microsatellite analysis of serum DNA in patients with oral squamous cell carcinoma. Oncol. Rep..

[67] Nunes D.N., Kowalski L.P., Simpson A.J.G. (2001). Circulating tumor-derived DNA may permit the early diagnosis of head and neck squamous cell carcinomas. Int. J. Cancer.

[68] Muhanna N., Di Grappa M.A., Chan H.H.L., Khan T., Jin C.S., Zheng Y., Irish J.C., Bratman S.V. (2017). Cell-free DNA kinetics in a pre-clinical model of head and neck cancer. Sci. Rep..

[69] Dietrich D. (2018). DNA methylation analysis from body fluids. Methods Mol. Biol..

[70] Weiss G., Schlegel A., Kottwitz D., König T., Tetzner R. (2017). Validation of the SHOX2/PTGER4 DNA methylation marker panel for plasma-based discrimination between patients with malignant and nonmalignant lung disease. J. Thorac. Oncol..

[71] Church T.R., Wandell M., Lofton-Day C., Mongin S.J., Burger M., Payne S.R., Castaños-Vélez E., Blumenstein B.A., Rösch T., Osborn N. (2014). Prospective evaluation of methylated SEPT9 in plasma for detection of asymptomatic colorectal cancer. Gut.

[72] Schröck A., Leisse A., De Vos L., Gevensleben H., Dröge F., Franzen A., Wachendörfer M., Schröck F., Ellinger J., Teschke M. (2017). Free-circulating methylated DNA in blood for diagnosis, staging, prognosis, and monitoring of head and neck squamous cell carcinoma patients: An observational prospective cohort study. Clin. Chem..

[73] Rodda A.E., Parker J., Spencer A., Corrie S.R. (2018). Extending circulating tumour DNA analysis to ultra-low abundance mutations: Techniques and challenges. ACS Sens..

[74] Pan B.T., Johnstone R.M. (1983). Fate of the transferrin receptor during maturation of sheep reticulocytes in vitro: Selective externalization of the receptor. Cell.

[75] Kalluri R. (2016). The biology and function of exosomes in cancer. J. Clin. Investig..

[76] Ruivo C.F., Adem B., Silva M., Melo S.A. (2017). The biology of cancer exosomes: Insights and new perspectives. Cancer Res..

[77] Zhang W., Xia W., Lv Z., Ni C., Xin Y., Yang L. (2017). Liquid biopsy for cancer: Circulating tumor cells, circulating free DNA or exosomes?. Cell. Physiol. Biochem..

[78] Abels E.R., Breakefield X.O. (2016). Introduction to extracellular vesicles: Biogenesis, RNA cargo selection, content, release, and uptake. Cell. Mol. Neurobiol..

[79] Van der Pol E., Boing A.N., Harrison P., Sturk A., Nieuwland R. (2012). Classification, functions, and clinical relevance of extracellular vesicles. Pharmacol. Rev..

[80] Wahlgren J., Karlson T.D.L., Brisslert M., Vaziri Sani F., Telemo E., Sunnerhagen P., Valadi H. (2012). Plasma exosomes can deliver exogenous short interfering RNA to monocytes and lymphocytes. Nucleic Acids Res..

[81] Rashed M.H., Bayraktar E., Helal G.K., Abd-Ellah M.F., Amero P., Chavez-Reyes A., Rodriguez-Aguayo C. (2017). Exosomes: From garbage bins to promising therapeutic targets. Int. J. Mol. Sci..

[82] Languino L.R., Singh A., Prisco M., Inman G.J., Luginbuhl A., Curry J.M., South A.P. (2016). Exosome-mediated transfer from the tumor microenvironment increases TGFβ signaling in squamous cell carcinoma. Am. J. Transl. Res..

[83] Park J.E., Tan H.S., Datta A., Lai R.C., Zhang H., Meng W., Lim S.K., Sze S.K. (2010). Hypoxic tumor cell modulates its microenvironment to enhance angiogenic and metastatic potential by secretion of proteins and exosomes. Mol. Cell. Proteom..

[84] Li L., Li C., Wang S., Wang Z., Jiang J., Wang W., Li X., Chen J., Liu K., Li C. (2016). Exosomes derived from hypoxic oral squamous cell carcinoma cells deliver mir-21 to normoxic cells to elicit a prometastatic phenotype. Cancer Res..

[85] Whiteside T.L. (2017). The effect of tumor-derived exosomes on immune regulation and cancer immunotherapy. Future Oncol..

[86] Theodoraki M.N., Yerneni S., Hoffmann T.K., Gooding W.E., Whiteside T.L. (2018). Clinical significance of PD-L1+ exosomes in plasma of head and neck cancer patients. Clin. Cancer Res..

[87] Ludwig S., Floros T., Theodoraki M.N., Hong C.S., Jackson E.K., Lang S., Whiteside T.L. (2017). Suppression of lymphocyte functions by plasma exosomes correlates with disease activity in patients with head and neck cancer. Clin. Cancer Res..

[88] Zlotogorski-Hurvitz A., Dayan D., Chaushu G., Salo T., Vered M. (2016). Morphological and molecular features of oral fluid-derived exosomes: Oral cancer patients versus healthy individuals. J. Cancer Res. Clin. Oncol..

[89] Rabinowits G., Bowden M., Flores L.M., Verselis S., Vergara V., Jo V.Y., Chau N., Lorch J., Hammerman P.S., Thomas T. (2017). Comparative analysis of microRNA expression among benign and malignant tongue tissue and plasma of patients with tongue cancer. Front. Oncol..

[90] Kai K., Dittmar R.L., Sen S. (2018). Secretory microRNAs as biomarkers of cancer. Semin. Cell Dev. Biol..

[91] Schafer C.A., Schafer J.J., Yakob M., Lima P., Camargo P., Wong D.T.W. (2014). Saliva diagnostics: Utilizing oral fluids to determine health status. Monogr. Oral Sci..

[92] Zhang Y., Sun J., Lin C.C., Abemayor E., Wang M.B., Wong D.T.W. (2014). The emerging landscape of salivary diagnostics. Oral Health Dent. Manag..

[93] Wong D.T. (2008). Salivary diagnostics: Amazing as it might seem, doctors can detect and monitor diseases using molecules found in a sample of spit. Am. Sci..

[94] Hu S., Li Y., Wang J., Xie Y., Tjon K., Wolinsky L., Loo R.R.O., Loo J.A., Wong D.T. (2006). Human saliva proteome and transcriptome. J. Dent. Res..

[95] Spielmann N., Wong D. (2011). Saliva: Diagnostics and therapeutic perspectives. Oral Dis..

[96] Spafford M.F., Koch W.M., Reed A.L., Califano J.A., Xu L.H., Eisenberger C.F., Yip L., Leong P.L., Wu L., Liu S.X. (2001). Detection of head and neck squamous cell carcinoma among exfoliated oral mucosal cells by microsatellite analysis. Clin. Cancer Res..

[97] Okami K., Imate Y., Hashimoto Y., Kamada T., Takahashi M. (2002). Molecular detection of cancer cells in saliva from oral and pharyngeal cancer patients. Tokai J. Exp. Clin. Med..

[98] Jiang W.W., Masayesva B., Zahurak M., Carvalho A.L., Rosenbaum E., Mambo E., Zhou S., Minhas K., Benoit N., Westra W.H. (2005). Increased mitochondrial DNA content in saliva associated with head and neck cancer. Clin. Cancer Res..

[99] Jiang W.W., Rosenbaum E., Mambo E., Zahurak M., Masayesva B., Carvalho A.L., Zhou S., Westra W.H., Alberg A.J., Sidransky D. (2006). Decreased mitochondrial DNA content in posttreatment salivary rinses from head and neck cancer patients. Clin. Cancer Res..

[100] Bahar G., Feinmesser R., Shpitzer T., Popovtzer A., Nagler R.M. (2007). Salivary Analysis in oral cancer patients: DNA and protein oxidation, reactive nitrogen species, and antioxidant profile. Cancer.

[101] Yuvaraj M., Aruna P., Koteeswaran D., Tamilkumar P., Ganesan S. (2015). Rapid fluorescence spectroscopic characterization of salivary DNA of normal subjects and OSCC patients using ethidium bromide. J. Fluoresc..

[102] Principe S., Hui A.B.Y., Bruce J., Sinha A., Liu F.F., Kislinger T. (2013). Tumor-Derived exosomes and microvesicles in head and neck cancer: Implications for tumor biology and biomarker discovery. Proteomics.

[103] Sharma S., Gillespie B., Palanisamy V., Gimzewski J.K. (2011). Quantitative nano-structural and single molecule force spectroscopy biomolecular analysis of human saliva derived exosomes. Langmuir.

[104] Langevin S., Kuhnell D., Parry T., Biesiada J., Huang S., Wise-Draper T., Casper K., Zhang X., Medvedovic M., Kasper S. (2017). Comprehensive microRNA-sequencing of exosomes derived from head and neck carcinoma cells in vitro reveals common secretion profiles and potential utility as salivary biomarkers. Oncotarget.

[105] Momen-Heravi F., Trachtenberg A.J., Kuo W.P., Cheng Y.S. (2014). Genomewide study of salivary microRNAs for detection of oral cancer. J. Dent. Res..

[106] Park N.J., Zhou H., Elashoff D., Henson B.S., Kastratovic D.A., Abemayor E., Wong D.T. (2009). Salivary microRNA: Discovery, characterization, and clinical utility for oral cancer detection. Clin. Cancer Res..

